# Inception of the Indian Digital Health Mission: *Connecting…the…Dots*

**DOI:** 10.1002/hcs2.67

**Published:** 2023-10-09

**Authors:** Gerard Marshall Raj, Sathian Dananjayan, Neeraj Agarwal

**Affiliations:** ^1^ Department of Pharmacology All India Institute of Medical Sciences (AIIMS) Bibinagar Hyderabad Telangana India; ^2^ Hospital Management Information System (HMIS) All India Institute of Medical Sciences (AIIMS) Bibinagar Hyderabad Telangana India; ^3^ School of Computer Science and Engineering Vellore Institute of Technology Chennai Tamil Nadu India; ^4^ Department of Community and Family Medicine All India Institute of Medical Sciences (AIIMS) Bibinagar Hyderabad Telangana India

## Abstract

The purpose of the National Digital Health Mission (or more precisely, the Ayushman Bharat Digital Mission) is to promote and facilitate the evolution of the National Digital Health Ecosystem in India. The Health Facility Registry, the Healthcare Professionals Registry, and the Unified Health Interface are the major components of the proposed system—which is intended to be a co‐operative federated architecture with optimal interoperability provision coupled with authorized access.

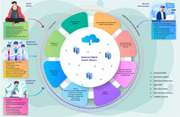

**Abbreviations**
ABDM: Ayushman Bharat Digital Mission
EHR: Electronic Health Records
EMR: Electronic Medical Records
HFR: Health Facility Registry
HPR: Healthcare Professionals Registry
NDHB: National Digital Health Blueprint
NDHM: National Digital Health Mission
PHR: Personal Health Records
UHI: Unified Health Interface

## 1 NATIONAL DIGITAL HEALTH MISSION (NDHM): THE BEGINNING

Ever since the National Health Policy, 2017 was released, the drive to digitalize the Indian healthcare system has been given more importance, in line with the United Nations' Sustainable Development Goals (SDGs). The Policy emphasizes the implementation of digital health in all levels of care—primary, secondary, and tertiary—to maintain the continuity of patient care. The application of digital technology tools and their integration in all facets of healthcare services is expected to improve the functional efficiency and outcomes of the Indian healthcare system [1].

The National Digital Health Blueprint (NDHB) was placed in the public domain after relevant stakeholder comments were incorporated [2]. The NDHB lays the foundation for how to proceed with the digitalizing process throughout the country [3]. More specifically, the proposed NDHM aims to break the existing internal barriers in the digital healthcare system. The National Health Authority (NHA) [4] is the apex body entrusted with the mandate for implementation and management of the NDHM across the country. The NDHB envisages the following 5‐year action plan: planning and stabilizing the NDHM (Year 1); establishing the prerequisite infrastructure (Year 2); execution (Year 3); analytics and innovation (Year 4); and sustenance and research (Year 5) [3].

In 2018, the National Institute for Transforming India (NITI Aayog) proposed digitalizing the Indian healthcare system through the National Health Stack (NHS) [5]. The NHS was formulated with the following key foundational components of the national digital health initiative: National Health Electronic Registries, a Coverage and Claims platform, a Federated Personal Health Records (PHR) Framework, a National Health Analytics Framework, and others. The NHS was orchestrated in such a way that the national digital infrastructure would be utilized by all parties—central and state governments, and public and private players. The multiple components of the NHS were later transformed and configured into the NDHM.

Healthcare in India, one of the most populous countries, requires connectedness among its different participants to sustain both essential and vital healthcare activities across the nation. Despite the intensification of the digitizing process in the health sector—like in any other industry—this process was primarily fragmented, and sharing of healthcare information across stakeholders was highly restricted, if not impossible at times [6].

**Figure 1:**
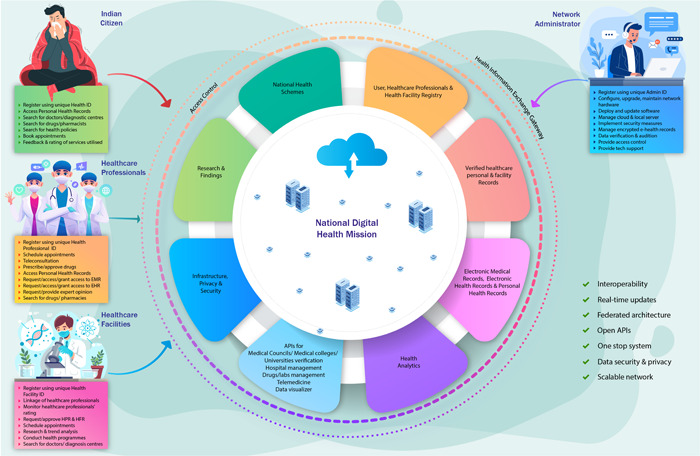
National digital health mission.

However, the proposed digital ecosystem would be more transparent, connecting healthcare providers (doctors), healthcare seekers (patients), and healthcare facilities (hospitals, clinics, laboratories, pharmacies). Accordingly, the three different domains are the Health Facility Registry (HFR), the Healthcare Professionals Registry (HPR), and the Unified Health Interface (UHI) (Figure 1) [7, 8].

The HFR and HPR are the twin core building blocks of the NDHM. The HFR is a comprehensive and all‐inclusive data repository of healthcare facilities, including hospitals, clinics, diagnostic laboratories, pharmacies, health and wellness centers, mobile vans, and ambulances. The HPR encompasses healthcare professionals from all domains and all systems of medicine, including doctors, dentists, nursing professionals, paramedical staff, laboratory technicians, physiotherapists, midwives, community health workers, dieticians, and nutritionists. Each facility must designate a facility manager who can enroll and register their facility. Once registered, a Health Facility ID (FID) is generated [7, 8]. As of April 2023, more than 200,000 health facilities have been registered in the HFR.

Healthcare professionals can link their Healthcare Professional ID (HPID) with their affiliated facility. To generate the HFID and HPID, certain information is mandatory, whereas other information is optional. For some information, verification is required, such as the registration license number of a physician, to be verified by the concerned medical councils, and educational qualification, to be verified by the concerned universities or medical colleges [7, 8]. As of April 2023, more than 160,000 healthcare professionals have been registered in the HPR.

Along similar lines with the present unified payment interface for monetary transactions, the planned UHI shall pave the way for the bidirectional flow of digital health service requests between end‐users and health service providers on an open platform. Broadly, the UHI gateway works through the following sequential components: from service discovery, service booking, service fulfillment, and financial settlement, to post fulfillment. Consultation bookings, ambulance bookings, availability of intensive care beds, enquiries about laboratory services, and house visits for sample collection are some of the digital health services envisioned [7, 8].

Under the UHI, individual patient health records would be managed through three different health information management schemes, namely, Electronic Medical Records (EMR) maintained in a particular health facility; Electronic Health Records (EHR) maintained across multiple doctors or facilities; and Personal (longitudinal) Health Records (PHR) maintained at the individual/patient level [3]. A Unique Health Identifier (UHID) or personal health identifier would also be created for uninterrupted service. As needed, there is also provision for cloud storage of health records in so‐called “Health Lockers” similar to the currently existent DigiLocker [7, 8].

These three prongs of NDHM were moderated and consolidated following due consultation with all stakeholders through a series of public webinars before finalization [8, 9].

The most lingering part of any digital system is its virtual existence and, consequently, its data security and data privacy; accordingly, the Health Data Management Policy of the NDHM was launched and subsequently updated. This policy upholds the importance of data protection and acts as the guiding document with minimum standards for an optimal‐cum‐advanced National Digital Health Ecosystem (NDHE) [10]. The NDHB was established based on the principle of “Zero Trust Architecture.” The proposed cloud storage, Health‐Cloud (H‐Cloud), would be monitored through both Security Operations Center and Privacy Operations Center [7]. The ultimate purpose is to make the available EMR‐EHR‐PHR data more usable [11] and stakeholder‐friendly [12], yet confidential [13]. Though the current system would be functioning entirely without restrictions, its individual autonomy would be still maintained with a co‐operative federated architecture (Table 1) [3, 5, 7].

**Table 1:** Features of National Digital Health Mission in comparison to the prevailing system.

Pre‐NDHM	Proposed NDHM
Isolated and functions in silo (Platform‐centric model)	Integrated and interoperable—breaking barriers (Open‐source software products and standards)
Centralized and sometimes, even decentralized architecture	Federated architecture Regional‐level State‐level District‐level Facility‐level
Fragmented and disrupted provision of healthcare	Continuity of care at all levels – primary, secondary, and tertiary – public and private
Interrupted and complex health governance	Improved efficiency, effectiveness, and accountability in health service delivery
Contemplation on hidden charges	Transparency in pricing of services rendered
Previous Health Facility Registries No real‐time updates Fragmented Not complete Lack of confidentiality and transparency	Health Facility Registry (HFR) Dynamic—provision for real‐time updates Universal—covers all systems of medicine and also both public and private organizations Comprehensive—includes all relevant information (basic, financial, civil and medical infrastructure) Data verified and audited regularly
Previous Registries Less representative ∘ Predominantly doctors of modern medicine Less participatory	Healthcare Professionals Registry Encompasses all healthcare professionals ∘ Doctors (from all systems of medicine) ∘ Dentists ∘ Nursing professionals ∘ Allied healthcare professionals ∘ Miscellaneous health workers Voluntary‐yet‐conducive for all stakeholders ∘ Medical Councils ∘ Medical Colleges/Universities ∘ Employer
Electronic Health Records (EHR) are not easily accessible whenever required	Demand‐driven, consent‐based, authenticate, and secure access to Electronic Health Records (Unified Health Interface)
Discrete and multiple EHRs (application‐centric)	Longitudinal, sequential, and standardized EHRs (citizen‐/patient‐centric)
Health Identifier is facility‐specific	Universal, unique, and secure Personal Health Identifier [Ayushman Bharat Health Account (ABHA) number]
Healthcare providers exist as mere health information generators	Healthcare providers act as Health Information Providers (HIPs) also referred to as Health Data Fiduciaries Healthcare providers can also act as Health Information Users (HIUs)
Only healthcare providers can generate health data	Incorporation of individual/patient generated data in the EHR is possible
Multiple heath Apps with no transfer of content across parties, even when needed	Multiple front‐end Apps with provision for authorized transfer of data. However, they can neither download individual patient data nor act as data repositories.
Each government health program/scheme has its own exclusive ID which leads to multiple IDs for each user	Government health programs/schemes can be accessed through a single Health ID (ABHA number)
Each member in a family has discrete Health IDs	Proposal for linkage of Health IDs of all members in a family

During the unprecedented COVID‐19 pandemic, the practical and clinical relevance of technology in delivering healthcare services—including the ArogyaSetu app, Co‐WIN, and e‐Sanjeevani, to name a few—was very much appreciated. The success of the Ayushman Bharat–Pradhan Mantri Jan Arogya Yojana scheme relies on its robust IT‐enabled system linking nearly 500 million beneficiaries. Some other examples of government‐managed health applications include NIKSHAY, an online tuberculosis patient monitoring application; the Integrated Disease Surveillance Program module of the Integrated Health Information Platform, a digital decentralized state‐based surveillance system; and the Mera Aspatal (My Hospital) patient feedback system.

The underlying principles of NDHM are to “capture data once and use many times,” to “move from silos to systems” by transcending isolated disparate electronic services to more integrated digital health services, and to act as a “single source of truth.” Leveraging the “JAM trinity” of Jan Dhan Yojana, Aadhaar and Mobile number can also fuel the process of digitalizing the Indian health system [7].

## 2 NDHM: THE PATH AHEAD

The importance of continuity of care has been much deliberated across all countries, irrespective of economic backgrounds [14, 15]. For this continuity of care to materialize optimally in a nation, adequate support is necessary to ease the passage of medical information between different areas of the country [16]. The major hurdle in accomplishing this exchange and usage of medical information is organizational [17]; hence, the onus lies with the health management authority of the concerned regions. This all‐inclusive pattern of the proposed NDHM would be beneficial at all levels from primary to advanced care settings and in both public and private segments—the evidence of which is appreciated in the real‐world Indian context with regard to utilization of EHR [18]. Furthermore, the negative impact of medical informational discontinuity is strongly felt among Indian doctors, particularly in clinical decision‐making [19].

In 2005, the World Health Assembly urged its member countries to implement eHealth services through which equitable, affordable, and universal access to healthcare services could be delivered. Accordingly, more than 120 countries have developed strategies and policies to strengthen eHealth services. In the subsequent World Health Assemblies (in 2013 and 2018), the impetus was shifted to national and global eHealth strategies—spreading the boundaries of the digital health initiative to all areas [20].

According to data from the World Economic Forum, there is a perceptible increase in digital health worldwide [21]. Broadly, the digital health market is larger in the Americas and Europe compared with those in Asia, the Middle East, or Africa, which translates to a direct correlation between per‐capita Gross Domestic Product and the digitalization of the healthcare system. However, there can be intraregional differences, as in Europe: Denmark, England, and Sweden are relatively better placed with regard to progress in digitalization of health compared with Ireland, Switzerland, and the Netherlands [21].

Even high‐income countries are confronting numerous obstacles in implementing the national digital health systems [22]. Hence, like any other national programs, the NDHM must be periodically monitored and evaluated for its expected outcomes [23]. The MEASURE Evaluation's Health Information System Strengthening Model provides guidance in the establishment and follow‐up of health information systems, particularly in low‐ and middle‐income countries. This model works in the four key areas, namely, the human element (workforce), the enabling environment (planning, implementation, and maintenance), information generation (operationalization), and performance (measurement of outcomes). Along with these key areas, the model also incorporates contextual factors, which vary by country [24].

In India, which has notably diverse, dense, and disproportionate distribution of populations and healthcare facilities, digitizing, compiling, and moderating health‐related data are indeed a significant task. However, if the processes continue as planned, the fruits of this noble pursuit of demand‐ and consent‐driven seamless transfer of health data will be far‐reaching, positively reflecting on all aspects of the health sector, if not the entire general economy of our country. More importantly, both providers and seekers of healthcare would become more empowered with timely and precise access to health information, which could have positive implications on the health status of every Indian. Eventually, overall public health is bound to improve with this strategic use of health care data [25]. Because the digital technologies are already in hand in India, the policy regulations (governance), infrastructure development, and capacity‐building are the only remaining necessities [26]. Hence, some of the expected challenges in implementing the Digital Health Mission could be nontechnological, including organizational, physical, political, social, cultural, and ethical bottlenecks [27].

Indian community medicine experts have provided possible solutions for the broad challenges in implementing the NDHM [28]. Some of their anticipated challenges were creating awareness and educating all players, reaching out to less‐privileged populations, manpower engagement and professional training, monetary constraints and sustainability, and data security matters. Other possible hurdles in adopting the NDHM model may relate to IT infrastructure (connectivity), digital literacy (understanding and knowledge), transformation inertia (application and functional issues), and cooperation from all stakeholders (social acceptability) [23].

Digital India is embracing four pillars, namely, the price of the device, the digital connectivity, the cost of data, and the idea of “digital first”. With the recent launch of 5 G services in India [29]—reportedly the fastest 5 G roll‐out in the world, deployed in 200,000 sites covering 700 districts in 8 months—the IT infrastructure has gained the necessary momentum to overcome obstacles to NDHM implementation.

The initial lag in the number of HFR and HPR registrations has been compensated for by a marked increase in HFR and HPR registrations following mass media campaigns. Additionally, the NHA has also announced the Digital Health Incentive Scheme to increase adoption by means of extending monetary bonuses for adopting the NDHM system.

The experience of countries such as Korea and the Netherlands in integrating national digital healthcare data into national health information systems could be an early learning exposure for India in overcoming possible obstacles [26]. Nevertheless, in the longer term, this Indian model of digital healthcare system can be a forerunner for other countries with similar economic and demographic backgrounds.

The establishment of the NDHM would not only strengthen the healthcare delivery services but also greatly reinforce other healthcare sectors such as health surveillance, health literature, and health education, knowledge, and research. The first building principle of the NDHE*—*“to think big”—is partially achieved by the promulgation of the NDHM, and this may make it easier to succeed in the other two principles, namely, “to start small” and “to scale fast.” Furthermore, in accordance with the above building principle, implementation of NDHM is planned in a phased manner with Phase 1 being a roll‐out in Union Territories, Phase 2 being expansion of services to other states, and Phase 3 being a nationwide roll‐out [7]. This phased establishment of the NDHM reflects positively on the sustainability of the national digital health system.

The NDHM was later rechristened as the Ayushman Bharat Digital Mission (ABDM) with the 14‐digit unique Ayushman Bharat Health Account (ABHA) Number as the UHID equivalent and the ABHA Mobile Application as the PHR equivalent. As of April 2023, nearly 380 million ABHA numbers have been generated and more than 262 million health records of Indian citizens have been digitized and linked with their ABHA number. Furthermore, as of March 2023, the ABHA‐based “Scan and Share” service has been utilized by more than 1 million patients for hassle‐free time‐saving outpatient registrations.

As stated by the Prime Minister during the launch of the ABDM, “Our brothers and sisters living in the remotest villages of the country will get better health facilities, through the launch of this mission today, we will go one step further in improving their lives.” [30] Hopefully, with the implementation of the Indian Digital Health Mission, we are on track to achieve this goal.

## AUTHOR CONTRIBUTIONS

**Gerard Marshall Raj:** Conceptualization (lead); methodology (lead); project administration (lead); writing—original draft preparation (lead); writing—review and editing (lead). **Sathian Dananjayan:** Methodology (supporting); project administration (supporting); visualization (lead); writing—review and editing (supporting). **Neeraj Agarwal:** Supervision (supporting); writing—review and editing (supporting).

## CONFLICT OF INTEREST

Authors declare no conflict of interest.

## ETHICS STATEMENT

This article does not contain any studies with human or animal participants.

## INFORMED CONSENT

Not applicable.

## Data Availability

Data sharing is not applicable to this article as no data sets were generated or analyzed during the current study.
